# Exploring the Immunological Mechanisms Underlying the Anti-vascular Endothelial Growth Factor Activity in Tumors

**DOI:** 10.3389/fimmu.2019.01023

**Published:** 2019-05-09

**Authors:** Rodrigo Barbosa de Aguiar, Jane Zveiter de Moraes

**Affiliations:** Department of Biophysics, Universidade Federal de São Paulo, São Paulo, Brazil

**Keywords:** vascular endothelial growth factor, bevacizumab, angiogenesis, Fc receptors, immune-modulation, immunity

## Abstract

Several studies report the key role of the vascular endothelial growth factor (VEGF) signaling on angiogenesis and on tumor growth. This has led to the development of a number of VEGF-targeted agents to treat cancer patients by disrupting the tumor blood vessel supply. Of them, bevacizumab, an FDA-approved humanized monoclonal antibody against VEGF, is the most promising. Although the use of antibodies targeting the VEGF pathway has shown clinical benefits associated with a reduction in the tumor blood vessel density, the inhibition of VEGF-driven vascular effects is only part of the functional mechanism of these therapeutic agents in the tumor ecosystem. Compelling reports have demonstrated that VEGF confers, in addition to the activation of angiogenesis-related processes, immunosuppressive properties in tumors. It is also known that structural remodeling of the tumor blood vessel bed by anti-VEGF approaches affect the influx and activation of immune cells into tumors, which might influence the therapeutic results. Besides that, part of the therapeutic effects of antiangiogenic antibodies, including their role in the tumor vascular network, might be triggered by Fc receptors in an antigen-independent manner. In this mini-review, we explore the role of VEGF inhibitors in the tumor microenvironment with focus on the immune system, discussing around the functional contribution of both bevacizumab's Fab and Fc domains to the therapeutic results and the combination of bevacizumab therapy with other immune-stimulatory settings, including adjuvant-based vaccine approaches.

## Introduction

The role of VEGF in driving tumor angiogenesis has made it an attractive target for therapeutic interventions, being bevacizumab, an FDA-approved humanized monoclonal antibody against VEGF, the most promising of them (1). Although these therapeutics were originally designed to control blood-vessel formation, increasing evidences point to their additional immunoregulatory role. In this mini-review, we uncover a more complete picture of the immunological changes induced by VEGF-targeting agents, specifically bevacizumab, in the tumor microenvironment (TME). We discuss the functional contribution of bevacizumab's Fab and Fc domains to the tumor immune landscape and outline the therapeutic potential of combining bevacizumab with other immune-stimulatory agents.

## The Immunotherapeutic Role of an Angiogenesis Targeted Agent

The vascular endothelium represents a barrier that lines the vessel compartment and regulates the access of blood components to the surrounding tissue. In tumors, however, this barrier is found corrupted. It determines a tortuous and disorganized vessel network with low pericyte coverage and high vascular permeability, contributing to install an immunosuppressive milieu (2, 3).

VEGF inhibitors, particularly bevacizumab, have been found to restore tumor blood vessel structure to normal, a process called vessel normalization (4). The “normalized” tumor vasculature results in increased tumor blood perfusion, higher pericyte coverage and reduced areas with sluggish blood flow, leading to enhanced influx of leukocytes into tumor parenchyma (5). On this topic, strong correlations were found between increased tumor-infiltrating lymphocytes—such as CD4^+^ and CD8^+^ T cells—and the vascular normalization imposed by VEGF pathway inhibitors (6, 7). The higher hydrodynamic force applied to endothelial wall may have a role on that, having in mind that a minimum level (above 0.5 dyn/cm^2^) of wall shear stress—that is, the parallel pressure exerted by the blood flow in the endothelial cell lining (5)—is required for enhanced endothelial cell expression of selectin family members, cell-surface molecules involved in leukocyte rolling in vessel wall (5, 8). On the other hand, the adhesion molecule content on the tumor blood vessel wall is also regulated by the local VEGF activity. Endothelial cell exposure to VEGF was found to hamper the expression of ICAM-1/2, VCAM-1, and CD34 molecules, all of them related to trans-endothelial cell migration and influx of leukocytes into the tumor parenchyma (9, 10).

Combined, these are structural and molecular characteristics of the TME, whose regulation affects the tumor vascular network and potentially participates in the bevacizumab-induced tumor recruitment of immune cells. Using a metaphor, the break imposed by VEGF inhibitors in the endothelial physicochemical barrier allows combat troops—here represented by the immune cells—access more easily the enemy territory—the tumor.

But the relationship between bevacizumab and the immune system is not only summarized by such indirect effects. In fact, the inhibition of VEGF also interferes directly in the activation and modulation of the immune response within the TME. In addition to vascular normalization, the pharmacological blockade of the VEGF/VEGFR axis can enhance the recruitment, trafficking and activation of CD8^+^ T-cell response in solid tumor models (9, 11, 12). Similarly, the expression levels of VEGF were found associated with decreased activation of CD8^+^ T and T_H_1 cell response on colorectal tumors (13), and the VEGF-enhanced expression of inhibitory checkpoints on CD8^+^ T cells can be reverted by VEGF- and VEGFR-targeted agents (14).

Beyond the effects on T cells, VEGF signaling also mediates tumor-associated immunodeficiency by expanding inhibitory immune cell subsets, such as FoxP3^+^ regulatory T lymphocytes (Tregs) and myeloid-derived suppressor cells (MDSCs). Tada and colleagues reported recently that the treatment of advanced gastric cancer patients with ramucirumab–a fully humanized IgG monoclonal anti-VEGF receptor 2 (VEGFR2) antibody–not only increased CD8^+^ T-cell tumor infiltration, but also significantly reduced the frequency of CD45RA^−^ FOXP3^high^ CD4^+^ cells (effector regulatory T cells [eTreg]) in tumors. Ramucirumab was also found to overcome VEGF-induced eTreg proliferation *in vitro* (15). These findings are in line with experimental data showing that VEGF directly enhances Treg proliferation in tumor-bearing mice. Moreover, bevacizumab significantly reduces the percentage of Tregs in peripheral blood from cancer patients and inhibits *in vitro* tumor cell-increased Treg proportion in PBMC (16, 17). In regard to MDSCs, it was found that VEGF promotes the expansion of these cells, being the CD11b^+^ VEGFR1^+^ MDSC population decreased in the peripheral blood of renal cell cancer patients treated with bevacizumab (18). Tumor-infiltrating MDSCs are known to contribute to the local immune suppression by inhibiting T cell activity and inducing Treg expansion (19).

Dendritic cells (DCs) and tumor-associated macrophages (TAMs) are other major components of the immune system that may be impaired by VEGF-targeting therapies. DCs are antigen-presenting units that act as messengers between the innate and adaptive immune systems. VEGF inhibits the DC precursor differentiation and maturation into functional cells capable of presenting tumor antigens and stimulating an allogeneic T-cell response. DCs were found inversely correlated with VEGF serum levels (20). Also, experimental data showed that the VEGF-induced DC dysfunction is recovered by both anti-VEGF and anti-VEGFR2 antibodies (20–25). When looking at TAMs, known as prominent players of the cell repertoire that populates tumors, we face again with a chemoattractant role of VEGF. The signal conferred by this growth factor contributes to increase the number of TAMs within the tumor bed and, as expected, VEGF inhibitors impair that (26–28). Also, VEGF-exposed macrophages were described to express endothelial cell markers and to contribute to vascular mimicry (29).

The role of macrophages in tumors varies depending on the environment. Based on their distinct regulatory and effector functions within the tissue microenvironment, TAMs are often classified on two major categories: (i) M1, designating classically activated macrophages that arose in response to IFN-γ, a T_H_1 signature cytokine; and (ii) M2, referring to “alternatively” activated macrophages induced by T_H_2-type cytokines (specifically IL-4 and IL-13), although we currently know that such yin-yang nomenclature does not recapitulate the whole spectrum of macrophage phenotypes (30, 31). From a tumor perspective, this classification not only reflects the T_H_1-T_H_2 polarization of T cell's response (32, 33), but also the TAM phenotype within the tumor landscape: while M1 macrophages exert antitumor functions, the M2-polarized ones are oriented toward promoting tumor growth, angiogenesis and tissue remodeling. Most TAMs acquire M2-skewed functions in the TME (34, 35), which means that the increased tumor macrophage content imposed by VEGF stimulation may contribute, together with the previously mentioned cellular effects, to establish an immunologically permissive environment for tumor growth. Although these data reveal that anti-VEGF settings decrease the frequency of TAMs in tumors, the VEGF-macrophage relationship goes further. Accumulation of M2-polarized macrophages within the TME was found as an indicator of tumor resistance to anti-VEGF therapy (36, 37), being possible targets to be explored in therapeutic approaches aiming to surpass such resistance. The vascular mimicry is among the M2 macrophage's contributions to the tumor refractoriness to anti-VEGF therapy (38).

## Exploring the Other Side of VEGF-targeted IgG Antibodies

Reducing the bioavailability of VEGF with full-length IgG antibodies compromises not only the tumor vasculature, but also the frequency and phenotype of immune infiltrative cells in tumors, changing the local ecosystem. But that is only the antibody's Fab side of the story.

The structure arrangement of bevacizumab, as of all other full-length IgG antibodies, comprises three functional domains, identified based on the product of the immunoglobulin digestion by papain: two Fab arms, and a single Fc domain (39). While the Fab arms have the variable amino acid sequence responsible for the antibody binding to the target antigen—which is, in that case, VEGF–, the significance of the Fc portion of IgGs lies on its ability to mediate cellular responses through a Fc-specific transmembrane receptor for IgGs (FcγR).

FcγRs are present on the surface of most cells from the immune system (39, 40). The binding of Fc domain of IgG to those specialized receptors initiates downstream effector functions, which englobes the antibody-dependent cell-mediated cytotoxicity (ADCC), antibody-dependent cellular phagocytosis (ADCP), and complement-dependent cytotoxicity (CDC). And that list goes further.

Data published so far reveal that FcγRs, when activated, signal a number of functional cellular changes in the tissue ecosystem, which is not limited to the immune repertoire. Functional FcγRs detected on endothelial and tumor cells contribute to pathway activations, cell proliferation and adhesion (41–46). Moreover, the signaling transduced by these receptors can even interfere in the tumor vascular network, an effect experimentally demonstrated by Bogdanovich et al. Bevacizumab was found to inhibit angiogenesis via Fc-mediated signaling through FcγR in a VEGF-independent manner (47). Such angioinhibition does not depend on ADCC, APCD, or CDC, suggesting the role of other FcγR-triggered effector responses induced by the antibody. It has also been reported that infusion of IgGs in both mice and humans inhibits angiogenesis (48) and that bevacizumab is more effective than its Fab fragment version—available commercially as ranibizumab—for the control of vessel formation (49). It is noteworthy that Fc-mediated effects were found to be required for achieving the maximum therapeutic effect of neutralizing antibodies (50, 51).

With our current knowledge, we cannot summarize all the effects triggered by bevacizumab-FcγR complexes. However, it should be kept in mind that the effects are not limited to what has been described above. To note, the IgG-FcγR interaction potentially provides critical scaffolding to trigger adaptive immunity. Experimental and clinical data revealed that passively administered IgG antibodies engage Fc receptors on DCs to stimulate a long-lasting anti-tumor cellular immune response (51), what is termed as “vaccinal effect.” Upon IgG immune complex binding, DCs undergo maturation and enhance CD8^+^ T-cell adaptive immunity through their antigen presentation function, as well as prime a T_H_1 CD4^+^ T-cell response (51, 52). Although most of these data were found for rituximab, the “vaccinal effect” potentially contributes to the Fc moiety of bevacizumab in mediating an immune response targeting VEGF found on TME cells. This is a point that deserves to be explored. Also, it should be considered that the expression of Fc receptors in TME cells is not fixed, being subjected to local changes that occur throughout treatment regimens. An example is FcγRIIb, a Fcγ receptor family member whose expression can be upregulated by T_H_2-type cytokines, such as IL-10, IL-4, and TGF-β, and downregulated by T_H_1-type cytokines, such as IFN-γ (53).

Taken together, these data highlight the diversity and complexity of the effects triggered by Fc domain-FcγR complexes within the TME profile, which certainly affect the tumor outcome. The understanding of the FcγR-mediated immunomodulatory pathways in different environments and cell subsets within the tumor ecosystem may be essential to improve the therapeutic benefits of bevacizumab.

## Combining Bevacizumab With Immune Modulators to Enhance Anti-tumor Response

The dual effect of bevacizumab on remodeling both vasculature and immune components of tumors opens up an opportunity for exploring combinatorial therapies aiming to enhance T_H_1 immune response against tumors. And some of the initiatives in this way seem promising.

Hodi et al. investigated the combination of bevacizumab and ipilimumab, an anti-CTLA-4 neutralizing mAb, in patients with metastatic melanoma. The blockade of CTLA-4, a negative regulator of T-cell responses, by ipilimumab may augment the endogenous anti-tumor cellular immune response, leading to tumor cell death. Results revealed an increased infiltration of CD8^+^ and CD163^+^ cells in tumors from patients receiving both mAbs, compared to the observed in tumor samples from the ipilimumab-only group (54). This was accompanied by the concentrated CD31 staining detected at interendothelial junctions of tumor vessels from the bevacizumab-treated group (54), which evidences the vascular changes occurring during VEGF blockade. CD31 is a vascular adhesion glycoprotein known to influence lymphocyte extravasation (54, 55), and whose expression and distribution pattern may have contributed to the detected intratumoral leukocyte content. These results are compatible with the obtained in further studies from the same research group (56).

The functional significance of the increased CD163^+^ cell population found under bevacizumab-containing regimen is not clear. CD163 is identified as a scavenger receptor for hemoglobin-haptoglobin complexes (57), but also as marker for M2 macrophages (58). Perhaps the increased CD163^+^ cell infiltration is an indicative of an evasive mechanism, mediated by M2 macrophages, to the anti-VEGF therapy. Besides that, few studies have investigated the functions of CD163, whose expression is also detected in subsets of classical and monocyte-derived DCs (59, 60). It is not even possible to discard that the expression of CD163 is an immune response to the extravascular hemoglobin content, secondary to necrosis index, eventually increased in tumors from patients receiving the combinatory treatment. Extravascular hemoglobin is a known endogenous danger signal that induces M2-skewed macrophage influx and CD163-macrophage polarization (61, 62). Clinically, CD163^+^ cell infiltration has been associated to both good (63) and bad (64–68) prognosis.

Similar benefits have been found under therapeutic interventions targeting programed cell death ligand 1 (PD-L1; a suppressor of the immune system). It was recently demonstrated that anti-VEGF and anti-PD-L1 combination therapy increases CD4^+^ and CD8^+^ cell infiltration in tumors and synergistically improves treatment outcome, compared to the obtained with each monotherapy (69). An ongoing clinical trial (ClinicalTrials.gov, trial identifier NCT01633970) is also investigating that. Moreover, even initiatives aiming to reprogram the M2 TAM-dominated TME have been put on the table, considering the described relationship between tumor M2 macrophages and bevacizumab resistance. Experimental study showed that combinatory treatment with a colony-stimulating factor 1 receptor kinase (CSF1R) inhibitor reduces the M2 macrophage content within tumors and aids in overcoming adaptive resistance to the herein explored anti-VEGF antibody performance (70).

Another approach with potential to be considered is the combination of bevacizumab therapy with adjuvant-based vaccines that stimulate a T_H_1 response against tumors. Vaccine adjuvants represent an attractive tool to modulate the immune cell effector function, with some of them being classified as inducers of T_H_1 T-cell immunity. That is the case of toll-like receptor agonists, such as dextran-conjugated CpG oligodeoxynucleotides (71) and double-stranded RNAs (72, 73), whose application in vaccine formulations enhances tumor-specific T_H_1-polarized CD4^+^ T cells and CTL responses. Although combining bevacizumab with vaccination settings seems to be a promising way to enhance the anti-tumor effect, there is no report on that up to now, with the few works in this direction limited to the use of T_H_1-inducer adjuvants in anti-VEGF vaccines (74, 75). And even in these cases, the results are restricted to the detection of specific cellular immune responses, remaining the clinical benefits yet to be demonstrated. In fact, all the herein exposed combination initiatives are in their first steps and further works are needed to clarify the effects in the TME and to achieve an optimized therapeutic protocol.

## A Matter of Antigen Specificity: Bevacizumab Recognizes Another Biomolecule Beyond VEGF

It is becoming increasingly evident that both Fab and Fc IgG domains—the two sides of the same coin—play a role in changing the vascular and immune components of solid tumors. As outlined above, a wide array of regulatory functions within TME are driven by the bevacizumab's constant region (Fc), which was not initially expected when this antibody was first employed in therapeutic settings. Likewise, it was not expected that bevacizumab's Fab domain recognizes other biomolecules in addition to the one it is known to identify.

Muller and coworkers demonstrated that bevacizumab directly binds to and sequesters the macrophage migration inhibition factor (MIF) from the TME. This may be due to certain similarities detected between amino acid sequences 48–76 of MIF and 29–51 of VEGF, the latter of which covers residues implicated in the bevacizumab binding (76).

MIF is described as an important regulator of immune responses (77). Experimental data showed that MIF downregulation led to increased intratumoral IFN-γ-producing CD4^+^ and CD8^+^ T cells, higher number of activated DCs, and reduced prevalence of MDSC and Tregs within tumors (36, 78–80), just as detected following VEGF inhibition. In the same direction, the interference with the MIF signaling was reported to decrease M2 macrophage shift in melanoma (81) and in multiple myeloma (82) models. Similar polarization effect was also found in microglial cells under MIF inhibition (83). Despite these findings, MIF is not always described as a M2 phenotype inducer. Lower levels of MIF at the tumor edge of glioblastomas were showed both to increase the local macrophage population, mainly from bone marrow-derived cells, and to polarize these cells to a M2 phenotype (36), which suggests that different microenvironmental contexts may imply in different MIF effects on tumor-infiltrating immune cells.

Overall, the functional significance of bevacizumab's Fab domain includes the inhibition of MIF. However, it is important to note that the direct binding to MIF is just one of the demonstrated mechanisms by which bevacizumab inhibits the MIF's function. MIF expression is transcriptionally regulated by VEGF (36), then subjected to the reduced local VEGF bioavailability imposed by bevacizumab therapy.

## General Overview

**Figure 1:**
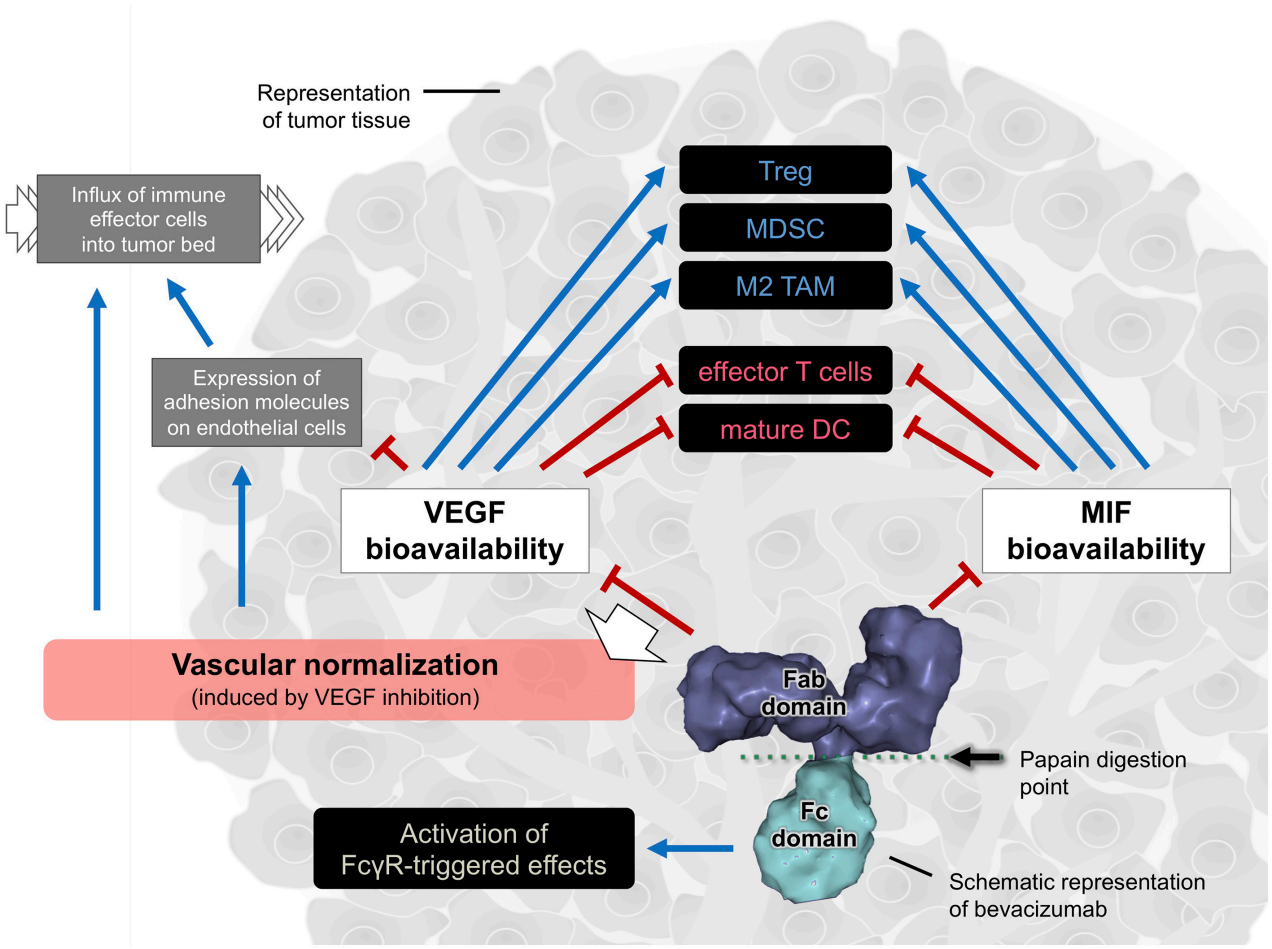
Cartoon summarizing the effects mediated by the bevacizumab's Fab and Fc domains in the tumor immune landscape. The vascular normalization, due to the blockade of excessive VEGF signaling in tumors, is one possible implication of bevacizumab therapy in tumors. The solid blue arrow lines indicate activation while the solid red hammer lines indicate inhibition. The black dashed line indicates inconclusive association (both activation and inhibition were reported). Surface model of an IgG (PDB entry: 1IGT) was used to represent bevacizumab. Image without scale.

Rather than just inhibiting angiogenesis, VEGF inhibitors have proved to regulate the immune response in tumors. The anti-VEGF antibody bevacizumab interferes in the composition and function of several immune cells within the TME, including T cells, TAMs, Tregs, MDSCs, and DCs. Bevacizumab was also found to trigger FcγR-mediated responses and to inhibit another immunoregulatory biomolecule beyond VEGF, which points out to the diversity of actions of this antibody in the tumor immune landscape. The herein described bevacizumab's immune-modulating effects are summarized on Figure 1. Overall, these data evidence that the therapeutic effects of anti-VEGF immunoglobulins reflect their multiple interactions with different elements that compose the tumor tissue. Understanding these effects is crucial to improve therapeutic effectiveness. That is a perspective beyond VEGF inhibition.

## Author Contributions

RA conceived and outlined the review. RA and JM contributed critically to the manuscript preparation.

### Conflict of Interest Statement

The authors declare that the research was conducted in the absence of any commercial or financial relationships that could be construed as a potential conflict of interest.

## Abbreviations

VEGF: vascular endothelial growth factor
TME: tumor microenvironment
Treg: regulatory T cells
MDSC: myeloid-derived suppressor cell
PBMC: peripheral blood mononuclear cell
DC: dendritic cell
TAM: tumor-associated macrophage
VEGFR: VEGF receptor
IFN-γ: interferon-γ
IL: interleukin
IgG: immunoglobulin G
FcγR: Fc-specific transmembrane receptor for IgG
ADCC: antibody-dependent cell-mediated cytotoxicity
ADCP: antibody-dependent cellular phagocytosis
CDC: complement-dependent cytotoxicity
TGF-β: transforming growth factor-β
CTLA: cytotoxic T-lymphocyte-associated protein-4
PD-L1: programed cell death ligand 1
CSF1R: colony-stimulating factor 1 receptor kinase
MIF: migration inhibition factor.
